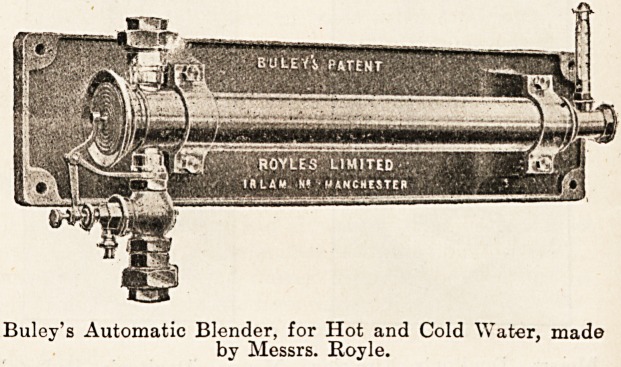# The Fitting and Furnishing of Wards and Operating Theatres

**Published:** 1907-07-27

**Authors:** 


### THE FITTING AND FURNISHING OF WARDS AND OPERATING THEATRES.

### LAVATORIES, WATER-CLOSETS, ETC.

Lavatory basins, sinks, and water-closet pans are all made of fire-clay, highly glazed, and it is the boast of the leading makers that they use no lead in the glaze, though this is not of much consequence so far as the final product is concerned. Flushing tanks for water-closets, slop-

Messrs. Doulton's Fire-clay Washdown Closet, arranged to stand clear of the floor, with hinged, oiled-teak seat.

Messrs. Doulton's Pedestal Fire-clay Closet, with oiled-teak, hinged seat.

sinks, etc., are made sometimes of glazed fire-clay, but more frequently of enamelled iron. All basins, pans, and tanks are constructed without corners, so that there may be no harbour for dirt, and hence for germs. In the great majority of cases also, basins, sinks and pans are fixed a few inches from the wall, the back surface facing the wall

Combined Draining Bench and Slop-sink, for cleaning Waterproofs and Bed-sanitas appliances.

being glazed, as the rest of the apparatus is, the object being to ensure that all parts are thoroughly cleansed and that it shall be easy to do so. There is a difference of opinion as to the benefits of the basins, etc., fixed away from the wall, and those built into the wall. Some hospital superintendents contend that the space between the basin and the wall is sometimes a harbour for dirt. All glazing must be so perfect that it does not chip, and the leading makers, Messrs. Doulton, and others, claim to have accomplished this without the aid of lead. The seats of the water-closets have given some anxiety to hospital superintendents, especially in the out-patient departments. In those departments in some hospitals the glazed fire-clay basins form the seats, this being the best method of preventing the deposit of germs. Another plan is, two pieces of wood shaped to cover the sides of the pan, are provided, and frequently changed, and a further extension of this is, a complete ring of wood is provided which again is frequently changed, the rings or segments of wood being inexpensive. In another arrangement by Messrs. Doulton the complete seat, of hard wood, is detachable.

### The Water Supply and Waste of Lavatories.

The main question in connection with the water supply is the control. Hot and cold are laid on to all lavatory basins, slop-sinks, etc., and the question is, how they shall most conveniently be mixed in the varying proportions required, and how the valves shall be manipulated. For the slop-sinks in the sanitary blocks of the

Messrs. Doulton's Operating-theatre Laboratory Basin, with separate Hot and Cold Water controlled by arm valves.

wards, screw-down valves are generally employed, but for lavatory basins of operating theatres there are three methods in use : the screw-down valves, corresponding to the usual domestic tap, valves controlled by levers worked by treadles, one each for hot, cold, and waste, and valves controlled by levers worked by the arm. Of these the latter find most favour, as they are arranged on the same lines as the ordinary tap. When the valve is opened to a certain extent by the arm valve it remains in that position till another movement of the arm alters it, the arm meanwhile being away from the valve; while with the treadle the foot has to be pressed down during the whole time the stream of water is required, to the extent of the flow required, and no surgeon is blessed with three feet; while it is awkward to operate two valves with one foot. The arrangement is, however, very ingenious and works well where the necessary foot manipulation can be given. A modification of the arm arrangement is a hand valve, worked with a short lever, having a loose handle. The handle when not in use is placed in a basin of sterilising liquid, and the surgeon takes it out of the basin, one handle sufficing for the two valves, places it on the end of the lever, turns on the water, and, when finished, returns the handle to the basin.

Mixing apparatus are very various for hot and cold water. In some forms the pipes from the two supplies are brought to a rose, the temperature of the resultant stream being regulated by the position of the two valves. In another form the same arrangement is employed, the two streams being united in one in place of being joined in a rose. In an apparatus made by Messrs. Doulton the mixing is performed by one valve, the temperature being raised or lowered by turning a single lever more or less, the construction of the valve inside being such that as the lever is

Messrs. Doulton's Operating-theatre Laboratory Basin, with Treadle Valves.

turned more or less hot water is allowed to pass. In another apparatus made by Messrs. Royle, of Irlam, near Manchester, that is in use at St. Thomas's Hospital, the hot and cold water is automatically blended to give the required temperature in the spray or stream. The hot and cold water separately enter a mixing chamber containing a thin copper tube with a diaphragm at one end. The apparatus having been set to furnish a stream or spray at any given temperature, the expansion and contraction of the air in the copper cylinder acts upon a diaphragm at one end which operates a bell crank motion, actuating a valve controlling the admission of the hot water.

The waste valves of basins and sinks are now all arranged in one with the overflow. Egress from the basin or sink is only by the orifice at the bottom, where the plug is in the domestic lavatory basin. In place of the plug there is a hollow cylinder of ebonite, or copper, standing in the orifice and open at the top. If the basin fills above the level of the top of the cylinder it flows down through the ordinary waste passage, by way of the inside of the cylinder. For emptying the basin the waste cylinder is pulled up just as the plug is. Ebonite cylinders answer very well, but are brittle, and at Guy's they have substituted copper.

### Cleaning and Drying Waterproof Sheets.

Waterproof sheets are usually cleaned on slabs or gratings attached to the slop-sinks, and are afterwards placed

Buley's Automatic Blender, for Hot and Cold Water, made by Messrs. Royle.

where they will dry gradually. In some hospitals they are placed before the ward fire at a sufficient distance to avoid injury to the india-rubber. In others there is a special drying department in the upper part of the building, where the sheets are spread out and are subjected to a gentle current of moderately-warmed air. In all cases the waterproof sheeting must be fully dried before being returned to its place in the ward. When dried the waterproofs are usually hung over horses provided for them covered by loose coverings of any kind that will keep the dust away. The horse is usually hinged so that it can be swung out clear of its coverings.

## Figures and Tables

**Figure f1:**
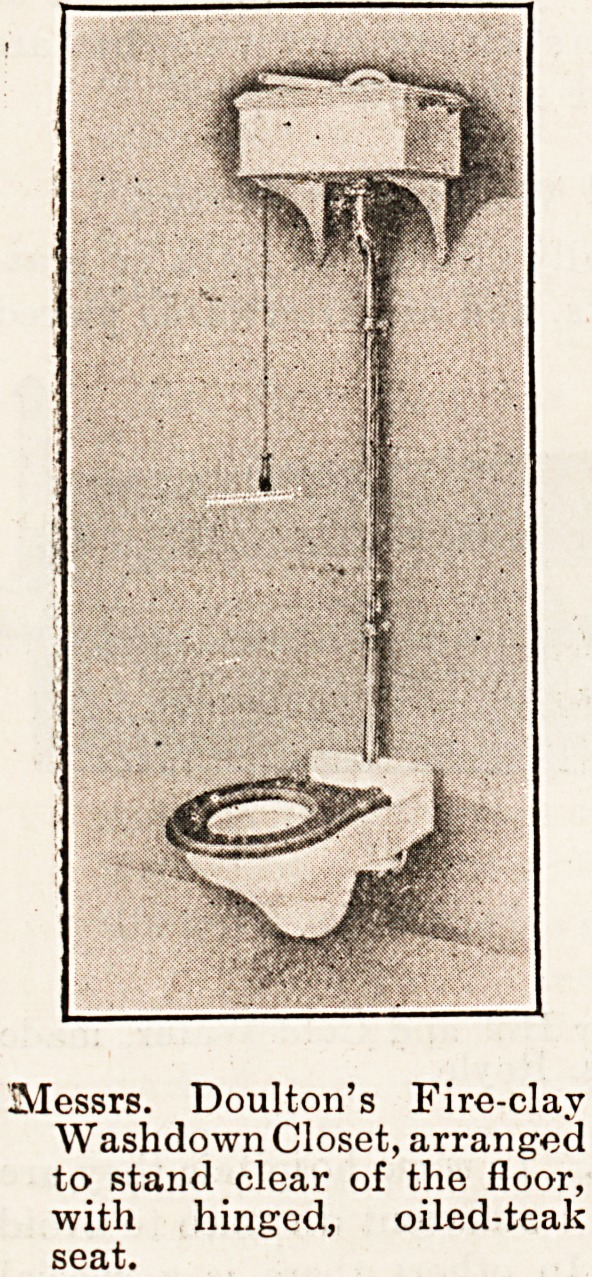


**Figure f2:**
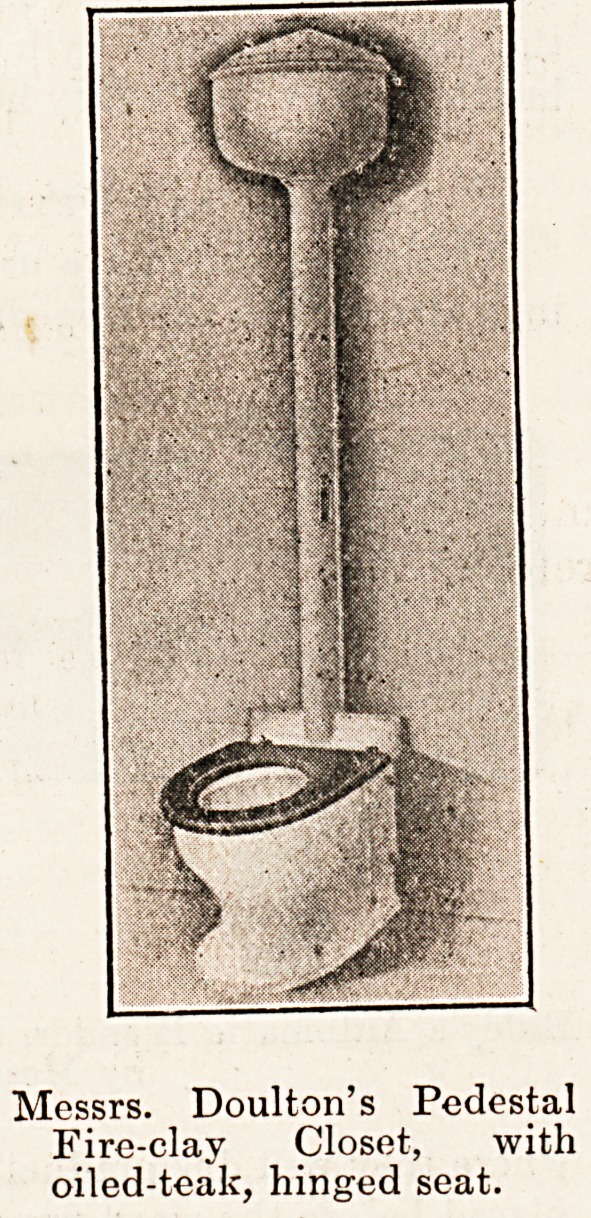


**Figure f3:**
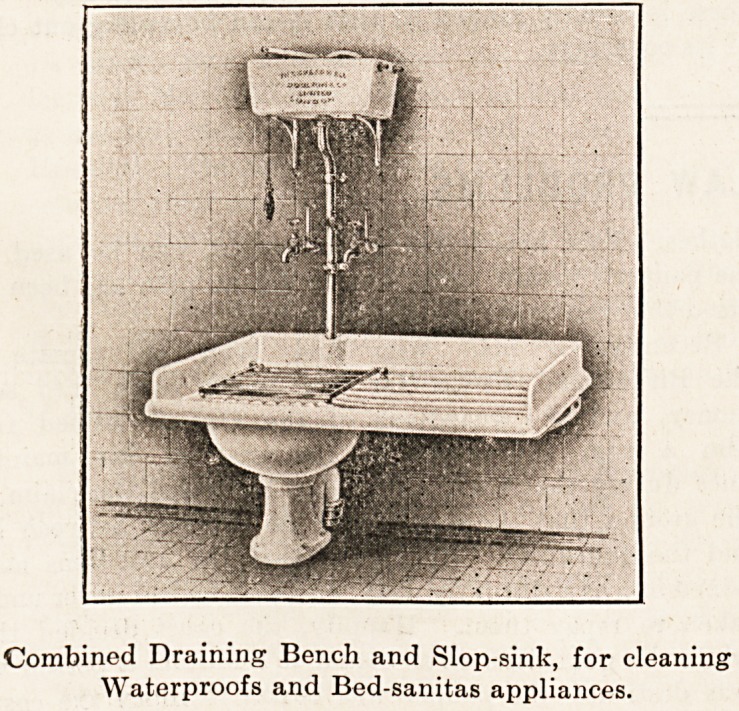


**Figure f4:**
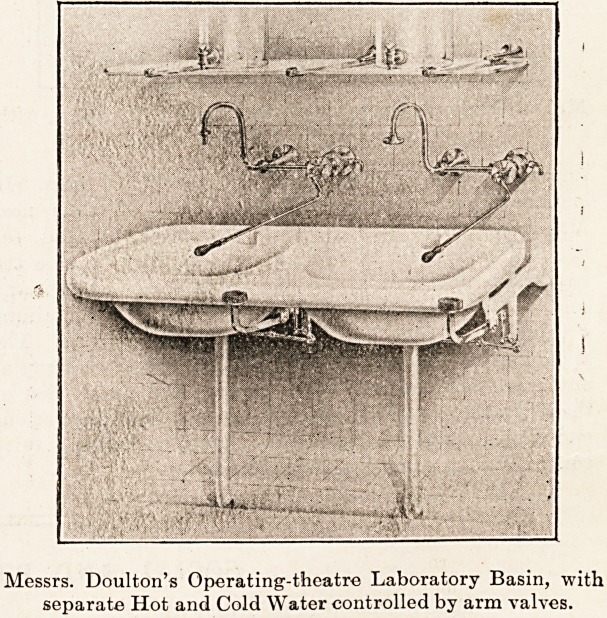


**Figure f5:**
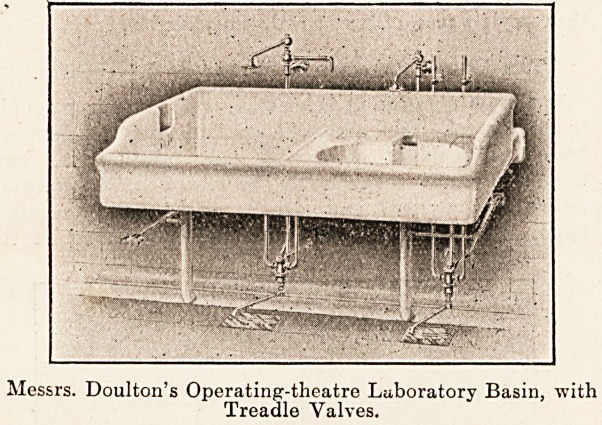


**Figure f6:**